# Quantification of respiratory depression during pre-operative administration of midazolam using a non-invasive respiratory volume monitor

**DOI:** 10.1371/journal.pone.0172750

**Published:** 2017-02-24

**Authors:** Luis N. Gonzalez Castro, Jaideep H. Mehta, Jordan B. Brayanov, Gary J. Mullen

**Affiliations:** 1 Neurology Department, Massachusetts General Hospital, Boston, Massachusetts, United States of America; 2 Anesthesiology Department, University of Texas Health Science Center at Houston, Houston, Texas, United States of America; 3 Research Department, Respiratory Motion Inc., Waltham, Massachusetts, United States of America; 4 Anesthesiology Department, Vidant Medical Center, Greenville, North Carolina, United States of America; University of Tübingen, GERMANY

## Abstract

**Background:**

Pre-operative administration of benzodiazepines can cause hypoventilation—a decrease in minute ventilation (MV)—commonly referred to as “respiratory compromise or respiratory depression.” Respiratory depression can lead to hypercarbia and / or hypoxemia, and may heighten the risk of other respiratory complications. Current anesthesia practice often places patients at risk for respiratory complications even before surgery, as respiratory monitoring is generally postponed until the patient is in the operating room. In the present study we examined and quantified the onset of respiratory depression following the administration of a single dose of midazolam in pre-operative patients, using a non-invasive respiratory volume monitor that reports MV, tidal volume (TV), and respiratory rate (RR).

**Methods:**

Impedance-based Respiratory Volume Monitor (RVM) data were collected and analyzed from 30 patients prior to undergoing orthopedic or general surgical procedures. All patients received 2.0 mg of midazolam intravenously at least 20 minutes prior to the induction of anesthesia and the effects of midazolam on the patient's respiratory function were analyzed.

**Results:**

Within 15 minutes of midazolam administration, we noted a significant decrease in both MV (average decrease of 14.3% ± 5.9%, p<0.05) and TV (22.3% ± 4.5%, p<0.001). Interestingly, the corresponding RR increased significantly by an average of 10.3% ± 4.7% (p<0.05). Further analysis revealed an age-dependent response, in which elderly patients (age≥65 years, n = 6) demonstrated greater reductions in MV and TV and a lack of compensatory RR increase. In fact, elderly patients experienced an average decrease in MV of 34% ± 6% (p<0.05) compared to an average decrease of 9% ± 6% (p<0.05) in younger patients.

**Conclusions:**

We were able to quantify the effects of pre-operative midazolam administration on clinically significant respiratory parameters (MV, TV and RR) using a non-invasive RVM, uncovering that the respiratory depressive effect of benzodiazepines affect primarily TV rather than RR. Such respiratory monitoring data provide the opportunity for individualizing dosing and adjustment of clinical interventions, especially important in elderly patients. With additional respiratory data, clinicians may be able to better identify and quantify respiratory depression, reduce adverse effects, and improve overall patient safety.

## Introduction

Benzodiazepines are commonly used in Monitored Anesthesia Care (MAC) in an attempt to pre-operatively reduce anxiety and provide anterograde amnesia.[1–3] Benzodiazepines are known to cause a reduction in respiratory effort, an undesired effect of their depressant action on the central nervous system. This can lead to hypoventilation, defined as a decrease in minute ventilation (MV) often referred to as respiratory compromise or respiratory depression. Over time, decrease in MV (Low MV) can lead to hypercarbia and/or hypoxia, secondary indicators often used in clinical studies as surrogates of respiratory depression since direct ventilation measurements were not available. Midazolam, a water soluble benzodiazepine, with a rapid onset and shorter duration of action than diazepam, has become a preferred agent for the management of pre-operative anxiety and induction of anesthesia.[4] While the exact dose-dependent relationship between midazolam (and other benzodiazepines) and respiratory depression has not been characterized, previous work suggests that the risk for adverse respiratory events increases with an increase in dose and is synergistically exacerbated by opioids and other anesthetic agents.[5]

Through the pre-operative use of benzodiazepines, patients are often placed at risk for respiratory complications even before their operations begin.[6] Such complications include an overall decrease in ventilatory response, a decrease in the mouth-occlusion pressure response to CO_2_, hypercarbia leading to respiratory acidosis, and apnea.[5] The extensive use of benzodiazepines may increase these risks, especially in older, frailer patients, where standard adult doses (common practice in some facilities in lieu of patient-specific dosing) can lead to a higher plasma concentration of drug and a higher effective dose.[7] Given the widespread use of benzodiazepines in the pre-operative setting, it is concerning that monitoring of respiratory parameters is generally postponed until the patient is in the operating room. A significant challenge in the pre-operative setting is the lack of continuous, non-invasive, real-time, respiratory monitoring that can provide reliable measurements of the adequacy of respiration.

Pre-operative respiratory monitoring is often limited to measurement of oxygen saturation. As a surrogate for respiration, pulse oximetry not only introduces a delay between the onset of respiratory depression and a low saturation alarm, but can also be subverted by the use of supplemental oxygen.[6] In fact, patients receiving supplemental oxygen can have normal SpO_2_ levels even in the presence of hypercarbic respiratory failure.[6] A better alternative, capnography, while clearly useful in intubated patients, has proven less effective in monitoring non-intubated patients intra- and post-operatively,[8] and its use has not been studied in the pre-operative setting. Research suggests that sidestream capnography may be more effective in monitoring non-intubated patients by using a fitted face mask or a sampling nasal cannula.[9] Yet, even under these ideal circumstances, the ability to reliably monitor respiration is limited. For instance, when the tidal volume drops below the sampling flow rate of the capnograph, such as during hypoventilation, the monitor compensates by entraining room air, thus falsely lowering the reading for the end-tidal carbon dioxide concentration, EtCO_2_.[10] In addition, in patients with underlying pulmonary disease, EtCO_2_ generally underestimates the level of arterial carbon dioxide, PaCO_2_, as measured from arterial blood sampling.[11] As hypoventilation is generally the root cause of perioperative hypercarbia and hypoxemia, low minute ventilation can be seen as the preferred early clinical indicator of a decline in respiratory function.[12]

A recently developed impedance-based, non-invasive respiratory volume monitor (RVM), which provides continuous, accurate measurements of minute ventilation (MV), tidal volume (TV) and respiratory rate (RR) has enabled reliable peri-operative respiratory monitoring in real time.[12,13] Previous studies with this monitor have shown its use in stratifying patients’ response to opioids in the post-operative period.[14] Here we used the RVM to quantify the effects of a 2 mg midazolam dose on the respiratory parameters of spontaneously breathing patients in the immediate pre-operative setting prior to Monitored Anesthesia Care (MAC). Our primary objective was to understand whether respiratory depression following benzodiazepines results in a decrease in tidal volume or a decrease in respiratory rate or both. In this process, we also identified and evaluated hypopneic and apneic events. As previously shown with opioids in the post-operative period, we hypothesized that the RVM could provide useful respiratory monitoring pre-operatively. Such RVM data may then be useful in developing a framework for stratifying patients who may be at an increased risk for respiratory depression secondary to benzodiazepines, allowing clinicians to more carefully titrate dosage for pre-operative use.

## Materials and methods

### 2.1 Patient cohort

Surgical patients at Vidant Medical Center (Greenville, NC), aged 18–99, were screened for their eligibility to participate in the current study. In order to be considered for admission into the study, patients were determined by an anesthesiologist as likely to remain spontaneously breathing during their operative course (either with sedation or general anesthesia). Fifty-eight spontaneously breathing adult patients undergoing elective surgery were entered into the study following written informed consent. Of the 58 patients, 30 (mean age 48 ± 17 years, range 20–80 years; average BMI 29.9 range 19-46kg/m^2^; 15 females, 14 orthopedic and 16 general surgery cases) received 2.0 mg of intravenous midazolam (Versed, Roche, Basel, Switzerland) at least 20 minutes prior to induction of anesthesia and were analyzed for this manuscript. The remaining 28 patients either (a) did not receive any midazolam pre-operatively (n = 9) or (b) received a 2 mg dose close to induction and as such we could not collect 20-minutes of data (n = 19). Note that, in our facility, the routine pre-operative care includes 2 mg of midazolam for every patient without contraindications and patient care was not altered for the purposes of this observational study.

### 2.2 Primary protocol

The study protocol was approved by the East Carolina University and Medical Center Institutional Review Board (#12–001260). The daily operating room schedule was used to screen prospective patients, based on type of case, and type of anesthetic technique requested by the operating surgeon. This study did not include patients scheduled to undergo thoracic surgical procedures so that uniform thoracic electrode placement could be maintained. No patients were excluded based on age, co-morbidities, or any other factors except for type of surgery as described above. Those that met the inclusion criteria were asked to participate. After obtaining written consent, an RVM (ExSpiron^™^, Respiratory Motion, Waltham, MA) was attached to each patient and calibrated using a Wright/Haloscale spirometer (nSpire Health, Inc., Longmont, CO), following standard padset placement[12,13] and it was used to monitor the patient’s respiratory function. Patients were additionally monitored using standard-of-care monitoring (IntelliVue^™^ Monitor, Philips Electronics North America, Andover, MA) and anesthesia equipment (Aespire S5 Advance, GE Healthcare, Waukesha, WI). As this study was observational, participating patients received standard care, standard pre-op sedation, and standard pain management not influenced by the RVM data. The clinical staff was blinded to the RVM measurements, and respiratory metrics were not displayed on the screen; however, waveforms were available to indicate proper function of the RVM.

### 2.3 Statistical analysis

Baseline values of the respiratory parameters (MV, TV, RR) were established during a quiet period, within 10 minutes prior to the administration of 2.0 mg of midazolam. To assess the full medication effect, data from the 15 minutes following drug administration was analyzed. The values reported throughout this manuscript are based on the time point of lowest sustained MV (at least 30 seconds, the minimum duration over which the RVM calculates respiratory parameters) within 15 minutes following the midazolam dose. The thirty patients were analyzed as a group and were also split into young (age<65 years, n = 24) and elderly (age≥65 years, n = 6) sub-groups.

Paired two-tailed t-tests were used to evaluate the effects of midazolam on MV, TV, and RR for the whole patient population, comparing baseline values (pre-midazolam) to post-midazolam values. Multivariate Analysis of Variance (MANOVA) was used to identify factors (height, weight, age, BMI, gender) that had significant effects on changes in the measured respiratory parameters. Un-paired t-tests were used to compare changes in MV, TV, and RR across age-stratified patient sub-groups. For all analyses, p<0.05 was considered significant. The analyses were performed in Matlab 2012b (MathWorks, Natick, MA).

## Results

**Fig 1:**
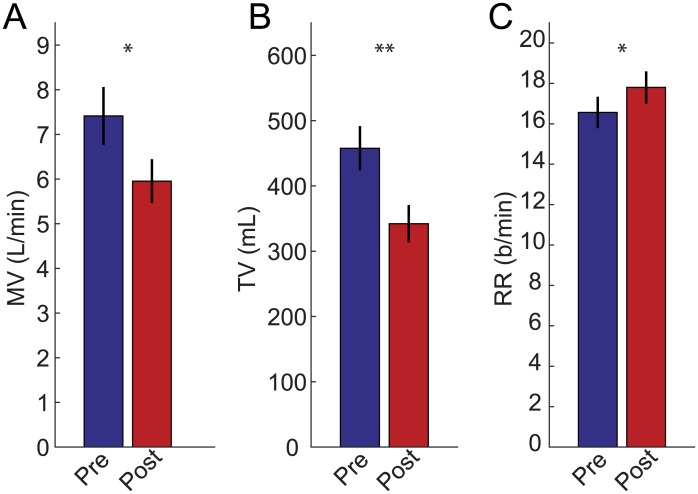
Average RVM measurements across the entire thirty patient cohort pre (left) and post (right) administration of 2mg of midazolam. (**A**) MV decreased significantly from 7.41 ± 0.62 L/min (mean ± SEM) to 5.95 ± 0.46 L/min, with an average decrease of 14.3% ± 5.9% (p<0.05). (**B**) Similarly, TV decreases significantly from 460 ± 30 mL to 340 ± 30 mL, an average decrease of 22.3% ± 4.5% (p<0.001). (**C**) In contrast, RR increased slightly from 16.6 ± 0.7 breaths per minute to 17.8 ± 0.7 breaths per minute, an average increase of 10.3% ± 4.7% (p<0.05).

All 30 patients (mean age 48 ± 17 years, range 20–80 years; 15 female; average BMI 29.9 kg/m^2^, range 19–46 kg/m^2^) included in our study received a standard 2 mg dose of midazolam in the pre-operative holding area at least 20 minutes prior to the induction of anesthesia. In order to assess the effects of the fixed midazolam dose on the patients’ respiratory drive, respiratory parameters (MV, TV, and RR) measured during the 10 minutes prior and the 15 minutes following the midazolam were analyzed. Across the entire cohort of 30 patients, we noted, after midazolam administration, a significant decrease in MV from 7.41 ± 0.62 L/min (mean ± SEM) to 5.95 ± 0.46 L/min, an average decrease of 14.3% ± 5.9%. p<0.05 (Fig 1A). Similarly, we measured a significant decrease in TV, from 460 ± 30 mL to 340 ± 30 mL, an average decrease of 22.3% ± 4.5%. p<0.001 (Fig 1B). Interestingly, the decrease in MV and TV was accompanied by a significant compensatory increase in respiratory rate from 16.6 ± 0.7 breaths per minute to 17.8 ± 0.7 breaths per minute, an average increase of 10.3% ± 4.7%. p<0.05 (Fig 1C).

**Fig 2:**
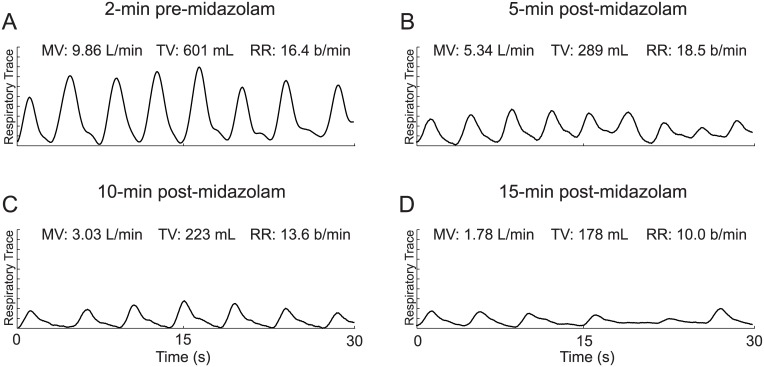
Thirty-second snapshots of RVM traces from a 66 y/o male patient (165 cm, 78 kg, BMI: 29 kg/m^2^) undergoing a left hip replacement surgery. These traces show a clear and immediate decrease in both MV and TV following midazolam administration, coupled with marginal and delayed decrease in RR. (**A**) Pre-midazolam values (2 min pre-dose) were taken as baseline. (**B**) Within 5 min of the dose, breathing was notably depressed with MV and TV effectively decreased by half, while RR marginally increased. (**C**) Ten minutes post-midazolam, MV and TV had decreased from pre-dose values by 69.3% and 62.9% respectively (MV: 9.9 to 3.0 L/min; TV 600 to 220 ml) while RR only decreased by 17.9% (16.4 to 13.6 b/min). (**D**) At 15 minutes post-midazolam, MV and TV had further decreased from pre-dose values by 81.9% and 70.4%, respectively (MV: 9.9 to 1.8L/min; TV: 600 to 180ml). RR finally shows a substantial decrease of 39.0% (RR: 16.4 to 10.0 breaths per minute).

**Fig 3:**
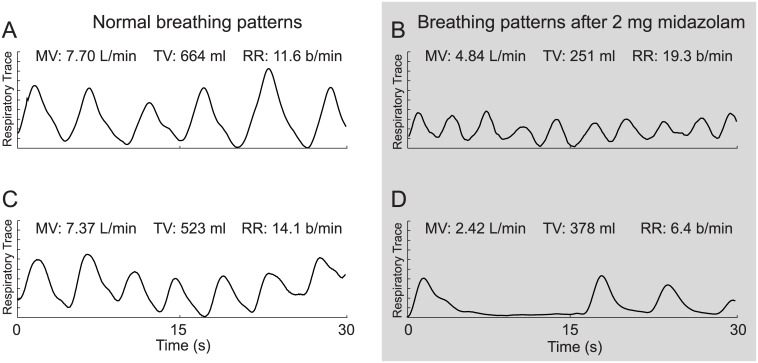
Examples of two different patterns of respiratory depression in two representative subjects. Panels A and B: 31 y/o male, 185 cm, 82 kg, BMI: 24 kg/m^2^. Panels C and D: 61 y/o female, 173 cm, 82 kg, BMI: 27 kg/m^2^. Comparison of traces before (**A** and **C**) and after (**B** and **D**) benzodiazepine administration shows that respiratory depression may manifest as both tachypnea with low TV (**B**) as well as intermittent apnea (**D**).

The response of a representative patient to a single 2 mg dose of midazolam is illustrated in Fig 2. Thirty-second snapshots of the respiratory trace, taken 2 min pre- and 5, 10, and 15 min post-midazolam administration, demonstrate substantial decreases in both MV and TV, coupled with an initial increase in RR. A comparison of panels A and B (2 min pre- vs. 10 min post-dose) shows that MV decreased by 46% (from 9.9 to 5.3 L/min) and TV decreased by 52% (from 600 to 290 mL), while RR increased by 13% (from 16.4 to 18.5 breaths/min). In fact, this initial decrease may have been desirable as this patient may have been anxious prior to surgery (hence the high MV). However, at 15 minutes post-midazolam dosing, MV and TV continued to decrease and were at approximately 20% of their respective pre-dose values and RR also showed a substantial decrease (Fig 2D), possibly indicating the patient had respiratory depression and may have required additional care. Fig 3 provides an additional example of respiratory traces and RVM measurements before (left) and after (right) midazolam administration, illustrating the different respiratory patterns manifested post-dose. While in the younger subject (31y/o male, Fig 3A and 3B) we see a reduction in TV, with a compensatory increase in RR to try to maintain minute ventilation levels, the older subject, a 61 y/o female (Fig 3C and 3D) not only shows a decrease in TV, but also a decrease in RR and regularity, leading to periods of apnea.

**Fig 4:**
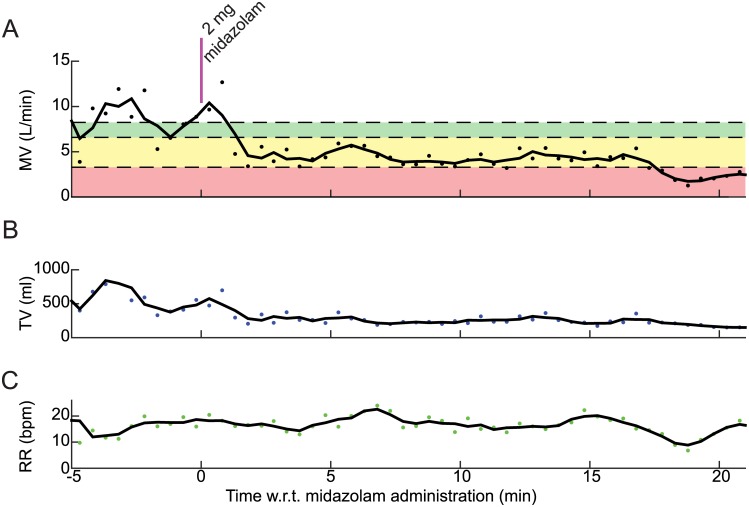
A time-lapse capturing one representative patient’s onset of respiratory depression during the 20 minutes following the administration of midazolam. Note that this is the same patient as in Fig 3A and 3B (31 y/o male). MV and TV decrease about 3–4 minutes after the dose and remain low as compared to pre-dose values, whereas RR increases and remains above baseline.

Whereas changes in MV and TV from one 30-second data acquisition period to the next may be quite subtle, the measurement trends over time can more easily demonstrate the substantial effect of the drug on respiratory status. Fig 4 shows the trends of RVM parameters over time profiling the onset and time course of midazolam-induced respiratory depression in a representative patient (Fig 3A and 3B). Fig 4A shows a clear downward trend in MV following the standard dose. This decrease in MV is primarily the result of a decrease in TV (Fig 4B) and is partially offset by a compensatory increase in RR.

**Fig 5:**
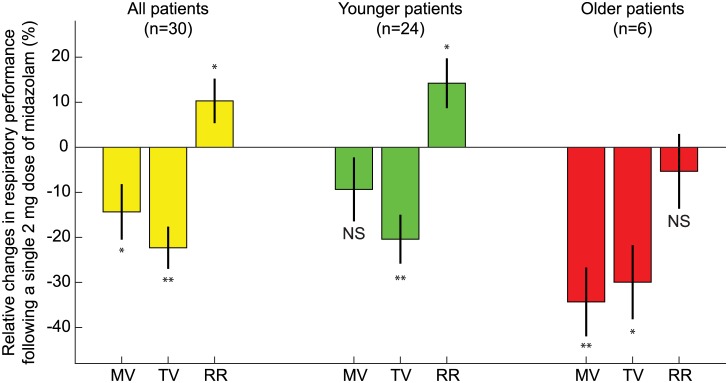
Age-specific analysis of respiratory depression following administration of 2mg of midazolam. Average changes in MV, TV, and RR following midazolam are shown for all patients (yellow), younger patients (green), n = 24, age 43 ±14 years, and older patients (red), defined as 65 years and older, n = 6, age 70 ±6 years. Both the young and elderly demonstrated significant decreases in TV (20% ± 5% and 30% ± 4% respectively, p<0.05 for both). In younger patients this decrease of TV was coupled with a significant increase in RR (14% ± 5%, p<0.05) leading to a marginal and not significant decrease in MV (9% ± 6%, p<0.05). In contrast, in the elderly patients RR did not increase, but in fact, slightly decreased (5% ± 4%, p>0.05) and the resulting MV was significantly lower, with an average decrease of 34% ± 6% (p<0.01).

Further analyses demonstrated no effect of height, weight, BMI, or gender (p>0.05 for all 4 of these factors) and a positive effect of age (p<0.05, 5-factor MANOVA). Sub-group analyses show a differential response to midazolam in participants aged 65 years and older. Not surprisingly, we found that the six elderly patients in our study (age≥65 years) were most susceptible to drug induced reductions in MV and TV, as summarized in Fig 5. Across the two subgroups and despite the limited sample size, we noted a significant age effect: MV in the elderly decreased significantly more than in the younger patients (34% ±6% vs. 9% ±6% p<0.05). Both the young (n = 24, age 43 ±14 years) and the elderly (n = 6, age 70 ±6 years) displayed significant decreases in TV (20% ± 5% and 30% ± 4% respectively, p<0.05 for both). Interestingly, in the younger patients this decrease of TV was coupled with a significant increase in RR (14% ±5%, p<0.05) leading to a marginal and not significant decrease in MV (9% ±6%, p>0.05). In contrast, in the elderly, RR following the dose of midazolam did not increase, but in fact, slightly decreased (5% ±4%, p>0.05) and the resulting MV was significantly lower, with a decrease of 34% ±6% (p<0.01).

## Discussion

In the present observational study we quantified the effects of the pre-operative use of midazolam on respiratory parameters. Across the entire cohort, administration of midazolam resulted in significant reductions in MV and TV, and a compensatory increase in RR. On average, MV and TV decreased by 14.3% and 22.3%, respectively, while RR increased by 10.3%. Interestingly, we noted a duality in the response to the same sedative between young and elderly patients. Young patients displayed a compensatory increase in RR coupled with a decrease in TV in an attempt to maintain MV, whereas elderly patients did not display a compensatory increase in RR. These findings confirm the inadequacy of relying on respiratory rate measurements alone to assess ventilation status which has been demonstrated in a variety of environments. While the elderly patients demonstrated a greater and potentially more dangerous response to midazolam, with decreases in both TV and RR, it is important to recognize that by modulating their RR more effectively, the younger patients generally demonstrated an adequate RR. Adequate RR may mask the presence of respiratory depression (Low MV) when RR measurements alone are used to assess respiratory status.

Previous studies have demonstrated that real-time MV measurements can be used to risk-stratify patients in the Post-Anesthesia Care Unit (PACU), where patients with MV less than 80% of their predicted MV prior to opioid administration had reductions in their MV to a critical level of less than 40% of predicted after opioid administration.[14] Similarly, we propose that it is feasible (with additional studies) to develop a risk-stratification framework based on patients’ responses to benzodiazepines in the pre-operative environment; identifying those in need of a modified anesthesia/sedation regimen and advanced monitoring post-operatively. Using real-time RVM measurements, clinicians will be able to individualize medication dosing and other therapeutic interventions. The need for patient-specific dosing is underscored by the wide variability in patient respiratory responses to the standard dose used of 2 mg midazolam that is used in our facility.

As in this study, clinicians often deliver standard doses of midazolam to their patients as part of an anesthetic regimen. Even with weight-based dosing, in an attempt to standardize the effective dose across patients, differences in gender, age, volume of distribution, chronic illness, and metabolic state[15] may still lead to a spectrum of responses, placing some patients at an elevated risk for adverse events. In fact, analysis of the RVM data in this small cohort revealed a significant age-dependent and weight-independent response to midazolam. These findings highlight the importance of patient monitoring and individualized treatment regimens. Pre-operatively identifying patients with an increased sensitivity to benzodiazepines may help protect them as they are more likely to encounter respiratory complications post-operatively in association with benzodiazepines often delivered for sedation or to relieve anxiety.

Identification of patients who exhibit suboptimal respiratory parameters after a small dose of benzodiazepine may also help define a group at greater risk of respiratory compromise from other medications used in the perioperative period, such as opioids. With the known synergistic effects of opioids and benzodiazepines on respiratory depression, this combination could be avoided or minimized in patients who have a dramatic response to pre-operative benzodiazepines. Such individualization of perioperative sensitivity to sedatives may help optimize patient safety across the continuum of care. Higher-risk patients should be identified and monitored more vigilantly, minimizing negative outcomes, while low-risk patients can be more rapidly advanced through the stages of recovery care. Such a preventative strategy would increase patient safety as well as decrease complications and their associated costs.

Although the use of pulse oximetry and capnography has been advocated in the literature,[16,17] as a means of assessing respiratory status in non-intubated patients, the efficacy of these modalities has been called into question.[6,8,17] SpO_2_ levels only begin to decline after respiratory decompensation has already begun,[6] serving as a delayed indicator of respiratory deficiencies. Reduction of alarm levels (e.g. to 80% saturation) in an effort to reduce false alarms only further delays recognition of respiratory depression.[18] Similarly, capnography and the reliance on EtCO_2_ is somewhat unpredictable, especially in non-intubated patients, and here too, false-positive alarms are also common, further reducing effectiveness.[8,19,20] As a leading indicator of respiratory compromise, MV measurement is the most effective way to unmask patient specific sensitivities to sedatives and narcotics, and can provide the foundation for individualized sedation and pain management protocols.

While the findings presented have important practical implications for the use of benzodiazepines pre-operatively, there are limitations to the study that are worth discussing. The cohort size was small, and although the magnitude of the effects was adequate to provide statistical significance, a larger study group may have revealed additional inter-dependencies, similar to the age-dependent relationship we observed. Second, the current study focused solely on the pre-operative course of care and for the purposes of this analysis, data collected during the operation or during the post-op recovery period were not considered. It would be beneficial to characterize interaction effects secondary to other agents administered intra-operatively and to identify long-term correlates of the observed effects of pre-operative benzodiazepine use during and post-surgery. Additionally, establishing each patient’s baseline respiratory performance during a period of normal, quiet breathing proved to be somewhat challenging given the busy environment of the pre-operative holding area. In general, patients were dealt with in a calm manner and appeared quite comfortable. None expressed significant anxiety. Fortunately, for each patient, we were able to identify a period of quiet and uniform breathing prior to the administration of midazolam which we used as our baseline, Nonetheless, the effect of stress associated with the frequently busy pre-operative holding area on patient respiration could have introduced a bias. Despite the fact that we were able to identify a period of quiet and uniform breathing for each patient prior to the administration of midazolam, in retrospect, it may have been preferable to measure the patients’ baseline respiratory performance in a less-dynamic environment.

Overall, this study provided additional evidence of the utility of RVM-based measurements in perioperative patient care. It demonstrated significant respiratory depression in some patients, especially the elderly, after a small standard dose of midazolam. RVM monitoring of such changes in respiratory status gives clinicians the opportunity to closely track the effects of benzodiazepines in non-intubated patients, allowing for more personalized care. Better characterization of respiratory status through the use of an RVM may also provide the basis for patient stratification pre-operatively. Such applications have great potential for preventing adverse respiratory depression and improving overall patient safety.
